# Dental caries status and its associated factors among schoolchildren aged 6–8 years in Hangzhou, China: a cross-sectional study

**DOI:** 10.1186/s12903-023-02795-5

**Published:** 2023-02-14

**Authors:** Zhi Chen, Junhua Zhu, Jing Zhao, Zhe Sun, Bing Zhu, Haiping Lu, Yuanna Zheng

**Affiliations:** 1 School/Hospital of Stomatology, Zhejiang Chinese Medical University, Hangzhou, Zhejiang China; 2 Hangzhou Center for Disease Control and Prevention, Hangzhou, Zhejiang China

**Keywords:** Oral health, Dental caries, Associated factors, Health behaviors

## Abstract

**Background:**

Dental caries prevalence reaches the first peak around 6 years old. It is necessary to take effective measures to prevent and treat caries at this stage. This study investigated the prevalence and associated factors of dental caries among schoolchildren aged 6–8 years in Hangzhou City, China.

**Methods:**

A cross-sectional survey was conducted in Hangzhou from October 2017 to May 2018. Oral health status of schoolchildren in 1st and 2nd grades of primary schools aged 6–8 years was examined by well-trained examiners according to the WHO criteria. Questionnaires about potential caries-related factors were distributed to their parents. ANOVA test and logistic regression were conducted for the statistical analyses (α = 0.05).

**Results:**

A total of 7959 pairs of schoolchildren and their parents were invited to participate, and 5595 were included in this study according to the eligible criteria (response rate: 70.3%). The overall prevalence and mean dmft/DMFT of dental caries in the investigated schoolchildren were 52.78% and 2.11. The prevalence and mean dmft/DMFT were 39.05% and 1.63 in the deciduous teeth, while these were 21.45% and 0.48 in the permanent teeth, respectively. For the first permanent molars (FPMs), the rate of fully eruption and pit and fissure sealing (PFS) were 79.09% and 6.60%, respectively. Multiple logistic regression showed that girl (OR = 1.12, 95% CI 1.01–1.25, *p* < 0.05), seldom or never brush teeth (OR = 2.36, 95% CI 1.08–5.44, *p* < 0.05), consuming sweet food or drink once or more time a day (OR = 1.14, 95% CI 1.00–1.29, *p* < 0.05; OR = 1.21, 95% CI 1.07–1.36, *p* < 0.05), dental visit experiences (OR = 1.58, 95% CI 1.35–1.86, *p* < 0.001) were positively and no tooth pain in the past 12 months (OR = 0.55, 95% CI 0.38–0.80, *p* < 0.05) were negatively associated with caries.

**Conclusions:**

Dental caries was prevalent among schoolchildren aged 6–8 years in Hangzhou, and was associated with gender, frequency of toothbrushing, sweet food or drink consuming, tooth pain and dental visit experiences. A large number of fully erupted FPMs did not receive timely PFS.

## Background

Dental caries is the most common chronic childhood disease [1]. It is the main cause of pain, and has an adverse effect on child’s eating patterns and sleeping quality [2]. Dental caries prevalence reaches the first peak around 6 years old [3], which is the initial of mixed dentition stage. Deciduous teeth are susceptible to caries, and the caries accumulates along with children’s growth [4]. Moreover, the first permanent molars (FPMs) begin to erupt or have already erupted during this period. FPMs are more vulnerable to caries than other permanent molars [5, 6]. In Chinese schoolchildren, mandibular FPMs had higher caries prevalence than maxillary ones, and the occlusal surface was the most common location for caries of all FPMs [7, 8]. Bacteria and food residues accumulates in the pits and fissures, dental plaque forms and matures undisturbed, and then the occlusal caries occurs [9]. Pit and fissure sealing (PFS) would be an effective measure to prevent occlusal caries [10].

The prevalence of dental caries and associated factors vary in different regions. According to a survey conducted in Shenzhen, China during 2016–2017, the caries prevalence among schoolchildren aged 6–8 was 56.59%, which was related to gender, type of schools, region, and Body Mass Index [11]. The caries prevalence among the same age group in Ryukyus, Japan during 2004–2005 was 81.5%, and household smoking might be associated with an increased prevalence of dental caries in schoolchildren [12]. In Riyadh, Saudi Arabia in 2015, the prevalence of dental caries was reported to be 83%, and 3 associated factors, including oral health behaviors and practices, child feeding practices, and dietary habits, were verified [13]. Therefore, in order to propose specific preventive measures, targeted investigation and analysis in local area are needed.

Hangzhou city is the capital of Zhejiang Province, China. It represents a rapid and diversified development economy in East China. In 2017, Hangzhou, with its gross domestic product (GDP) surging 8% year-on-year to 1.25 trillion yuan, ranked 10th among all cities in China. Permanent resident population has reached 946.8 million with an increase of 3.05% compared with 2016. The recent survey about dental caries status among schoolchildren aged 6–8 years in Hangzhou was from 2009 to 2011 [14]. Another study in the same age group was conducted across Zhejiang province during 2013–2017, but focused only on the FPMs [7].

As dental caries is a socioeconomic related disease, the rapid development of the city may bring about changes of dental caries status. Therefore, this study aimed to investigate the dental caries status among schoolchildren aged 6–8 years in Hangzhou and its associated factors. The results would provide a basis for formulating corresponding strategies and implementing interference programs to control the dental caries, and might improve oral health among these schoolchildren in future.

## Methods

This study was part of the first standardized oral health promotion program in China organized by Chinese Stomatological Association (CSA-2017-001).

### Sample selection

As it was not feasible to select students individually, a cluster-based sampling method was used in the sampling process, with the primary school acting as a cluster. The determination of sample size is calculated as $n=\frac{{z}_{{\upalpha }}^{2}p\left(1-p\right)deff}{{\updelta }^{2}}$. A design effect (deff) of 2.5, ${\upalpha }=0.05$ and a marginal error of 2.5% was considered. The expected prevalence of the dental caries was taken as 70.9% in accordance with 4th National Oral Health Survey (NOHS) in China [16]. According to enrollment policy issued by Hangzhou Education Bureau, the age of the schoolchildren in 1st and 2nd grades of primary school were 6–8 years. The expected average number of students in 1st and 2nd grades of each primary school was 150. It was estimated that a total of 31 primary schools were needed considering 70% response rate among students. Finally, a total of 32 primary schools were considered to account for any non-response in at most one primary school.

During the 2017–2018 academic year, 32 primary schools were randomly selected among 458 schools in Hangzhou city. Before survey, the written informed consents about the study were distributed to the parents of children from 1st and 2nd grades in those randomly selected schools. Non-Chinese children or children with non-Chinese caregivers or parents were excluded. Further, parents who were neither single, nor having psychiatric conditions or cognitive dysfunctions, were invited to answer the questionnaire [15]. Children who have completed the examination, with parents returned the completed questionnaires were eligible.

### Clinical examination

Clinical examination was conducted according to basic methods of oral health survey (5th edition) proposed by World Health Organization (WHO). During examination, the participants were required to sit on a mobile dental chair and were examined with mouth mirror and probe under artificial light. Caries of deciduous teeth and permanent teeth were assessed by using prevalence of caries, and mean dmft/DMFT respectively. Filling rate was calculated by dividing the number of filled teeth by the number of dmft/DMFT. The status of FPMs was further examined, including fully erupted or not, and sealed or not. All examiners were general dental practitioners with at least 3 years’ experience. Before survey, they were well-trained followed by a standardized calibration using various kinds of intraoral photographs of the children with target age. In the beginning of survey, each examiner was asked to check 10 schoolchildren for twice (1 day apart) to assess the intra-examiner reliability, and the results were compared with which given by the standard examiner to assess the inter-examiner reliability. Additionally, 5% of the investigated schoolchildren were randomly re-examined. Cohen’s KAPPA test verified that reliability and reproductivity of all examiners (kappa > 0.80).

### Questionnaire

Parents, who signed the written informed consents, were asked to complete a self-administered structured questionnaire, which mainly came from the 4th NOHS. It included (1) basic information like student id, name, gender, et al., (2) individual factors like toothbrushing habits, dietary habits, tooth pain, and dental visit experience, and (3) family factors like only one child or not, and parents’ education level. Parents were assessed regarding their knowledge and attitude towards oral health: nine items for the domain “knowledge” and four items for the domain “attitude”. The parents could obtain assistant whenever they met problems, like incomprehensible terminology. A pilot study was conducted with schoolchildren of the same age in a different school [15]. The repeatability of this questionnaire was convincing since no significant difference was found between the results of the pilot study and the main study.

### Statistical analysis

EpiData 3.0 was used to double entry and double checking the data of the oral examination records and questionnaires. Parents’ responses to oral health knowledge and attitude were scored as following: correct response to oral health knowledge and positive response to oral health attitude were scored 1, incorrect response to oral health knowledge and negative response to oral health attitude were scored 0, and the rest of responses were scored 0. The total score for each parent was calculated to represent parents’ oral health knowledge level and attitude, respectively. ANOVA test was applied to investigate the difference of mean dmft/DMFT by categorical variables. Univariate logistic regression analysis was performed to find the potential associated factors (*p* < 0.1) and those variables further included in the multiple logistic regression model. SPSS software (IBM SPSS Statistics 25, IBM) was used for data analysis with the level of statistical significance set at *p* < 0.05.

## Results

A total of 7959 schoolchildren aged 6–8 years from 32 primary schools in Hangzhou participated in the clinical oral examination. All their parents were invited to complete the questionnaire, and 5595 valid questionnaires were collected according to the eligible criteria. Therefore, 5595 pairs of schoolchildren and parents were included in this study, resulting in a response rate of 70.3% (5595/7959).

Table 1 presents the dental caries status of those schoolchildren. The overall prevalence and mean dmft/DMFT of dental caries were 52.78% and 2.11. For deciduous teeth, the prevalence, mean dmft, and filling rate were 39.05%, 1.63, and 40.98%, respectively. For permanent teeth, the prevalence, mean DMFT, and filling rate were 21.45%, 0.48, and 12.38%, respectively.

**Table 1:** The dental caries status of schoolchildren aged 6–8 years in Hangzhou, China (N = 5595)

	Schoolchildren with caries (n)	Prevalence of dental caries (%)	dt/DT		mt/MT		ft/FT		Mean dmft/DMFT	Filling rate (%)
			Mean	Constituent ratio (%)	Mean	Constituent ratio (%)	Mean	Constituent ratio (%)		
Deciduous teeth	2185	39.05	0.93	57.06	0.03	1.84	0.67	41.10	1.63	40.98
Permanent teeth	1200	21.45	0.41	85.42	0.00	0	0.06	12.50	0.48	12.38
Total	2953	52.78	1.34	63.51	0.04	1.90	0.73	34.60	2.11	34.53

*dt/DT* decayed teeth index for (deciduous + permanent), *mt/MT* missing teeth index for (deciduous + permanent), *ft/FT* filled teeth index for (deciduous + permanent), *dmft/DMFT* decayed, missing, and filled teeth index for (deciduous + permanent)

Table 2 shows the status of FPMs. The fully eruption rate of FPMs was 79.09%, while 63.58% of FPMs had already established the occlusion. However, the rate of PFS was relatively low, which was just 6.60% of the fully erupted FPMs.

**Table 2:** The status of first permanent molars (FPMs) in schoolchildren aged 6–8 years in Hangzhou, China

Index	Number of FPMs	Percentage (%)
Eruption stage		
Fully erupted with occlusion established	14,229	63.58
Fully erupted without the establishment of occlusion	3472	15.51
NOT fully erupted and were covered with a gingival flap	1993	8.91
The cusps just break through the gums	502	2.24
Not erupted	2184	9.76
Pit and fissure sealing of the fully erupted FPMs	1168	6.60

**Fig. 1:**
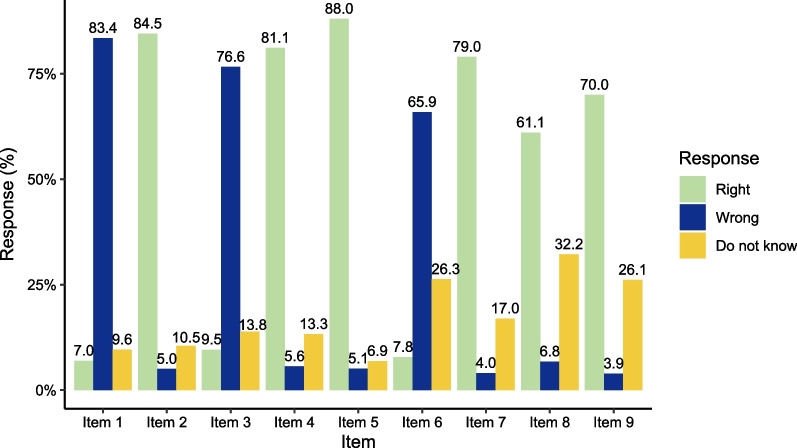
The responses of parents’ oral health knowledge. Item 1: It is normal for gums to bleed while brushing teeth; Item 2: Bacteria is one of the causes of gingivitis; Item 3: Brushing teeth does not help prevent gingivitis; Item 4: Bacteria causes dental caries; Item 5: Eating dessert is associated with increased dental caries risk; Item 6: Fluoride does not help to protect teeth; Item 7: Pit and fissure sealing can prevent caries; Item 8: Oral diseases will cause some certain systemic diseases; Item 9: Dental plaque is the main cause of caries and gingivitis. The response ‘wrong’ of item 1, 3, and 6 is the correct response, while the response ‘right’ of other items is the correct response. The response ‘right’, ‘don’t know’ and ‘wrong’ was scored 1, 0, and 0 respectively

**Fig. 2:**
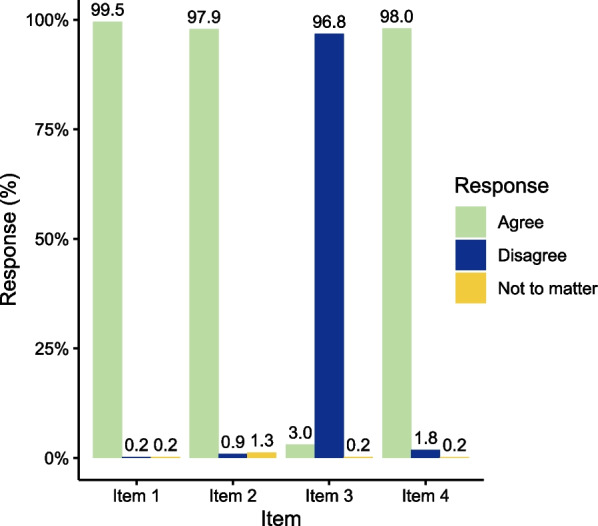
The responses of parents’ oral health attitude. Item 1: Oral health is very important to personal life; Item 2: A regular dental visit is important; Item 3: The quality of teeth is innate, and has little to do with personal behavior; Item 4: Preventing oral diseases depends on ourselves. The response ‘disagree’ of item 3 is the positive response, while the response ‘agree’ of other items is the positive response. The response ‘agree’, ‘disagree’ and ‘not to matter’, was scored 1, 0, and 0 respectively

Figures 1 and 2 show the responses of parents’ oral health knowledge and attitude respectively. For parents’ oral health knowledge, the correct rate of the questions related to “It is normal for gums to bleed while brushing teeth”, “Bacteria as the one cause of both gingivitis and dental caries”, and “eating dessert is associated with increased dental caries risk” were over 80%. The 2 questions with the lowest correct rate were related to the protection of fluoride (65.9%), and the association between oral diseases and some certain systemic diseases (61.1%). For parents’ oral health attitude, the responses of all questions were positive, and the rate were close to 100%.

As shown in Table 3, the mean dmft/DMFT of the investigated children was similar between different genders, soft drinking consuming status, beverage with sugar drinking status, parental oral health attitude, and whether the investigated child was one child or not (*p* > 0.05). Statistical difference in dental caries between different parental education levels were observed (*p* < 0.05). A significantly lower mean dmft/DMFT was observed in the bachelor and above degree for father (1.93) and mother’s (1.95) education than the other 3 subgroups. Schoolchildren who brushed teeth twice or more time a day had a mean dmft/DMFT as 1.71, which was significantly lower than schoolchildren who seldom or never brush teeth, 2.19 (*p* < 0.05). Consuming more dessert related to a significantly higher mean dmft/DMFT, 2.31, than consuming less dessert, 2.04 (*p* < 0.05). Schoolchildren who never visited the dentist had a significant lower mean dmft/DMFT, 1.71 (*p* < 0.001). And the mean dmft/DMFT of schoolchildren who always had toothache in the past 12 months was 2.81, while that of schoolchildren who never had a tooth pain in the past year was 1.65 (*p* < 0.001).

**Table 3:** Relationship of mean dmft/DMFT and the selected variables analyzed by ANOVA

Variable	Investigated schoolchildren (N, %)	Mean dmft/DMFT	*p* value
Gender			0.058
Boy	2942 (53)	2.04	
Girl	2653 (47)	2.18	
Only child			0.272
Yes	2519 (45)	2.06	
No	3076 (55)	2.14	
Father's education level			0.004*
Middle school and below	984 (18)	2.17	
High school	1356 (24)	2.21	
Junior college	1122 (20)	2.25	
Bachelor and above	2100 (38)	1.93	
Mother's education level			0.006*
Middle school and below	1154 (21)	2.11	
High school	1264 (23)	2.3	
Junior college	1238 (22)	2.14	
Bachelor and above	1904 (34)	1.95	
Tooth brushing frequency			0.047*
≥ 2 times a day	3457 (62)	1.71	
Once a day	1859 (33)	1.86	
Not every day	251 (4)	2	
Seldom or never	28 (1)	2.19	
Time since last dental visit			< 0.001*
Never	1264 (23)	1.71	
More than 1 year	1361 (24)	2.23	
Less than 1 year	2970 (53)	2.22	
Dessert			0.002*
< 1 time/day	4202 (75)	2.04	
≥ 1 time/day	1393 (25)	2.31	
Soft drinks			0.437
< 1 time/day	1829 (33)	2.07	
≥ 1 time/day	3766 (67)	2.13	
Beverage with sugar			0.149
< 1 time/day	2384 (43)	2.17	
≥ 1 time/day	3211 (57)	2.06	
Tooth pain in the past 12 months			< 0.001*
Always	124 (2)	2.81	
Occasionally	2352 (42)	2.64	
Never	2947 (53)	1.65	
Don't remember	172 (3)	2.1	
Parents’ oral health knowledge			0.011*
Score 0–3	925 (17)	2.33	
Score 4–6	4310 (77)	2.05	
Score 7–9	360 (6)	2.26	
Parents’ oral health attitude			0.802
Score 0–2	155 (3)	2.05	
Score 3–4	5440 (97)	2.11	

The significant difference (*p* < 0.05) of mean dmft/DMFT between the different levels of each variable was marked as asterisk (*)

Table 4 presents the logistic regression for the dental caries. Seven variables were selected as the potential associated factors of dental caries and entered into multiple logistic regression model. Girl (OR = 1.12, 95% CI 1.01–1.25, *p* < 0.05), seldom or never brush teeth (OR = 2.36, 95% CI 1.08–5.44, *p* < 0.05), consuming sweet food or drink once or more time a day (OR = 1.14, 95% CI 1.00–1.29, *p* < 0.05; OR = 1.21, 95% CI 1.07–1.36, *p* < 0.05), dental visit experiences (OR = 1.58, 95% CI 1.35–1.86, *p* < 0.001) were positively and no tooth pain in the past 12 months (OR = 0.55, 95% CI 0.38–0.80, *p* < 0.05) were negatively associated with caries of the investigated schoolchildren (*p* < 0.05). Although mothers’ education level was selected into the multiple logistic regression model, no statistical association had been found (*p* > 0.05).

**Table 4:** Logistic regression for the dental caries status

Variable	Univariate		Multiple	
	OR (95% CI)	*p*	OR (95% CI)	*p*
Gender				
Boy (ref)	–	–	–	–
Girl	1.14 (1.03, 1.27)	0.01	1.12 (1.01, 1.25)	0.04
Only Child				
Yes (ref)	–	–		
No	1.06 (0.95, 1.17)	0.32		
Father's education level				
Middle school and below (ref)	–	–		
High school	1.00 (0.85, 1.18)	0.99		
Junior college	1.07 (0.90, 1.27)	0.45		
Bachelor and above	0.90 (0.77, 1.05)	0.18		
Mother's education level				
Middle school and below (ref)	–	–	–	–
High school	1.23 (1.04, 1.44)	0.01	1.15 (0.98, 1.36)	0.09
Junior college	1.13 (0.96, 1.33)	0.13	1.03 (0.87, 1.22)	0.74
Bachelor and above	0.99 (0.86, 1.15)	0.91	0.91 (0.77, 1.07)	0.24
Tooth brushing frequency				
≥ 2 times a day (ref)	–	–	–	–
Once a day	1.36 (0.61, 3.17)	0.46	1.51 (0.66, 3.60)	0.33
Not every day	1.85 (0.86, 4.18)	0.12	1.97 (0.90, 4.54)	0.10
Seldom or never	2.18 (1.02, 4.92)	0.05	2.36 (1.08, 5.44)	0.04
Time since last dental visit				
Never (ref)	–	–	–	–
More than 1 year	1.88 (1.61, 2.20)	< 0.001	1.58 (1.35, 1.86)	< 0.001
Less than 1 year	1.85 (1.62, 2.11)	< 0.001	1.50 (1.29, 1.73)	< 0.001
Dessert				
< 1 time/day (ref)	–	–	–	–
≥ 1 time/day	1.23 (1.08, 1.38)	0.001	1.14 (1.00, 1.29)	0.04
Soft drinks				
< 1 time/day (ref)	–	–	–	–
≥ 1 time/day	1.16 (1.04, 1.30)	0.008	1.21 (1.07, 1.36)	0.002
Beverage with sugar				
< 1 time/day (ref)	–	–		
≥ 1 time/day	1.08 (0.97, 1.20)	0.17		
Tooth pain in the past 12 months				
Always (ref)	–	–	–	–
Occasionally	1.10 (0.76, 1.58)	0.62	1.07 (0.73, 1.55)	0.73
Never	0.52 (0.36, 0.74)	< 0.001	0.55 (0.38, 0.80)	0.002
Don't remember	0.93 (0.58, 1.49)	0.76	0.96 (0.59, 1.55)	0.86
Parents’ oral health knowledge				
Score 0–3 (ref)	–	–		
Score 4–6	0.92 (0.80, 1.06)	0.26		
Score 7–9	1.05 (0.82, 1.35)	0.68		
Parents’ oral health attitude				
Score 0–2 (ref)	–	–		
Score 3–4	0.99 (0.72, 1.37)	0.97		

The variables, whose *p* < 0.1 analyzed by univariate logistic regression, were involved in the multiple logistic regression model

## Discussion

The present study reported the latest dental caries status among schoolchildren aged 6–8 years in Hangzhou, China. In this study, the caries prevalence and mean dmft of deciduous teeth among the investigated schoolchildren in Hangzhou (39.05% and 1.63, respectively), were lower than the results in 2011 (58.4% and 3.41, respectively) [14]. The filling rate of deciduous teeth increased from 4.28% in 2011 [14] to 40.98% in 2018, and the increase was a 10 times. It indicated that the status of deciduous teeth among schoolchildren aged 6–8 years in Hangzhou improved significantly. This trend was contrary to the 4th NOHS, which revealed the caries prevalence and mean dmft of deciduous teeth increased considerable over the last 10 years [16]. In the 4th NOHS, the investigated children aged 3–5 years were from 31 provinces and municipalities in China. The caries prevalence of deciduous teeth reached 70.9%, while that was 39.09% in Hangzhou. The mean dt and its constituent ratio were 4.06 and 95.8%, which were significantly higher than the results of the present study (0.93, 57.06%). Meanwhile, the mean ft and its constituent ratio reported in NOHS were 0.17 and 4.0%, which were significantly lower than the results of the present study (0.67, 41.10) It could be attributed to the different populations surveyed. Significant differences may be related to Hangzhou's rapid economic development, and its GDP growth exceeding the national average level (6.9%). Oral health awareness and behavior of Hangzhou residents on protecting deciduous teeth had obvious improvement.

The caries prevalence and mean DMFT of permanent teeth among the investigated schoolchildren in Hangzhou in this study (21.45% and 0.48, respectively), were higher than the results in 2011 (7.1% and 0.15, respectively) [14]. The present result was consistent with the study in Zhejiang Province [7]. During 2013–2017, the caries prevalence on FPMs of schoolchildren aged 6–8 years was 20.4%, 25.3%, 24.5%, 27%, and 29%, respectively, showing an increasing trend [7]. From the composition ratio of the 3 indices, it can be seen that a large number of decayed teeth have not been filled. Compared with the previous study, the filling rate of permanent teeth decreased significantly, from 23% in 2011 [14] to 12.38% in 2018. It indicated that the caries status of permanent teeth at the beginning stage of mixed dentition of children in Hangzhou should arouse more attention.

In present study, fully eruption rate of FPMs (79.09%) in Hangzhou was similar to the average level in Zhejiang Province (78.7%) [7], and just 6.60% of the fully erupted FPMs in this study showed PFS. It may be related to two reasons. On the one hand, many parents (21%) still did not know or disagreed with the positive effect of PFS. On the other hand, PFS has been listed in Hangzhou public health project since 2010, and the PFS time of FPMs were when the children entered the 1st and 2nd grades of primary school. Considering that the best period for PFS to prevent caries should be when the crown has fully erupted and the caries have not yet developed [17], and the high fully erupted rate of FPMs among schoolchildren in 1st and 2nd grades observed in the present study, the unified PFS time implemented currently seemed not timely enough, which needs to be brought forward.

Six variables were identified as the associated factors of schoolchildren aged 6–8 years in Hangzhou. Although there was no significant difference of mean dmft/DMFT between girls and boys in the present study, the positive association of girl with caries was found, which was agreed with the previous studies [8, 11]. It may be related to the fact that girls consumed more sweets [18], and had earlier teeth eruption than boys [19]. Toothbrushing was an effective way to remove plaque and prevent dental caries. In consistent with previous studies [20, 21], as the frequency of brushing decreased, the caries increased gradually. Moreover, seldom or never brush teeth was found as a positive associated factor of caries in the present study. However, no effect of fluoride toothpaste on the prevalence of caries existed. Consuming too much sweet food and soft drinks was also associated with caries, supported by previous studies [22, 23]. Hangzhou cuisine belongs to Zhejiang cuisine, which contains much sugar. Meanwhile, with the rapid development of economy and increase of foreign diets, schoolchildren got more sweet food and soft drinks [24]. In present study, overwhelming majority of parents agreed that eating dessert is associated with increased dental caries risk. However, there were still many schoolchildren consumed much sweet food and soft drinks. Tooth pain and dental visit experiences were the other 2 important associated factors. Currently a common phenomenon in China was that more than half of schoolchildren who visited dentist was for treatment other than check-up [15]. No tooth pain in the past 12 months was negatively and having dental visit experience was positively associated with caries.

For the schoolchildren aged 6–8 years in Hangzhou, the significant influence of parents’ education level on the dental caries was revealed. The mean dmft/DMFT decreased with the increase of parents’ education level. Because parents with higher education level may have better oral health awareness or higher income to improve their living and health conditions than other parents, which were benefit for reducing their children’s dental caries [4, 25, 26]. However, the association of this variable with caries was not statistically verified in the present study. Similarly, although there was significant difference of mean dmft/DMFT of investigated children among parents with different levels of oral health knowledge, the variable was not identified as the associated factor of dental caries either. The present study found that parents had good oral health knowledge and positive attitudes generally, but some of them did not practice well. De Jong-Lenters et al. found that parenting practices and parent–child interactions had influence on the dental caries in children aged 5–8 years old [27]. Howenstein et al. revealed that authoritative parents, who were both high warmth and high control, were associated with less caries and better oral behaviors during their children’s first dental visit [28]. A study conducted in Japan clarified that poor parenting was associated with poor oral health behaviors and high prevalence of dental caries among children aged 6–7 years [29]. Knowledge-attitude-practice is one of the modes to change the human being’s health-related behaviors. For parents in Hangzhou, the way to improve the situation should be to educate them with personalized methods so that they would take action actually.

One limitation of the present study was that some important variables were not involved, like family income, parents’ career, schoolchildren’s oral health knowledge and attitudes, et al. Another limitation of the present study was that the affected surfaces of the teeth were not be recorded due to excessive workload. Although they would not influence the results of the dental caries, they were helpful to clarify potential associated factors and explain the findings. Meanwhile, through cross-section study, it was impossible to determine that the associated factors found in the study was the real cause of caries. Therefore, a well-designed longitudinal study should be conducted in future.

## Conclusions

Dental caries was prevalent among schoolchildren aged 6–8 years in Hangzhou, China. Gender, frequency of toothbrushing, sweet food or drink consuming, tooth pain and dental visit experiences were verified as the associated factors of dental caries. A large number of FPMs had fully erupted, and most of them were without timely PFS.

## Data Availability

The datasets used and analyzed during the current study are available from the corresponding author on reasonable request.

## Abbreviations

WHO: World Health Organization
OR: Odds ratio
CI: Confidence intervals
DMFT: Decayed, missing, and filled teeth index for permanent teeth
dmft: Decayed, missing, and filled teeth index for deciduous teeth
dmft/DMFT: Decayed, missing, and filled teeth index for (deciduous + permanent) teeth
FPMs: First permanent molars
PFS: Pit and fissure sealing
GDP: Gross domestic product
NOHS: National Oral Health Survey
